# Reliability and Validation of the PFIQ-7 and PFDI-20 in the Luganda Language

**DOI:** 10.1007/s00192-024-05866-5

**Published:** 2024-07-12

**Authors:** JaNiese Elizabeth Jensen, Michael Derrick Ngobi, Flavia Matovu Kiweewa, Julia Diane Fleecs, Ramya Vemulapalli, Haley Alaine Steffen, Linder Hagstrom Wendt, Jay Brooks Jackson, Kimberly Ann Kenne

**Affiliations:** 1 University of Iowa Carver College of Medicine, Iowa City, USA; 2 Makerere University-John Hopkins University Research Collaboration, Kampala, Uganda; 3 University of Iowa Institute for Clinical and Translational Science, Iowa City, USA; 4 Department of Pathology, Carver College of Medicine, University of Iowa, Iowa City, USA; 5 Department of Obstetrics and Gynecology, University of Iowa, 200 Hawkins Drive 31674 PFP, Iowa City, IA 52242 USA

**Keywords:** Disorders, Pelvic floor, Republic of Uganda, Translating, PFDI-20, PFIQ-7

## Abstract

**Introduction and Hypothesis:**

Pelvic floor disorders (PFDs) impact women worldwide and are assessed using instruments such as the Pelvic Floor Distress Inventory (PFDI-20) and Pelvic Floor Impact Questionnaire (PFIQ-7). There are no known valid PFD instruments in Uganda. This study’s purpose was to translate and test the reliability and validity of the PFDI-20 and PFIQ-7 in Luganda. It was predicted that these instruments would be reliable and valid to assess the presence and impact of PFD in parous Luganda-speaking women.

**Methods:**

The translated PFDI-20 and PFIQ-7 were administered to parous Luganda-speaking women and readministered 4–8 months after. The Pelvic Organ Prolapse Quantification (POP-Q) examination determined the presence of pelvic organ prolapse (POP) and a cough-stress test (CST) measured urinary leakage. Analysis was completed using Cronbach’s α co-efficient for internal consistency and Spearman’s correlation coefficients and Wilcoxon rank sum tests for construct validity.

**Results:**

Of the 159 participants, 93 (58.3%) had stage II POP or higher. The PFDI-20 and PFIQ-7 demonstrated minimal bother and impact on activities of daily living respectively. The Urinary Distress Inventory 6 (UDI-6) scores on the PFDI-20 showed a strong positive association with the presence of urinary incontinence. When PFD was defined by responses to symptom assessment, the translated PFDI-20 and PFIQ-7 could differentiate between individuals with and without PFD.

**Conclusions:**

The UDI-6 section of the PFDI-20 was found to be valid in Luganda. The PFIQ-7 and the entirety of the PFDI-20 were not found to be reliable or valid, likely because of the low prevalence of PFDs in the study population.

## Introduction

It is estimated that 25% of women in the United States (US) are affected by pelvic floor disorders (PFDs), which include anal incontinence (AI), urinary incontinence (UI), and pelvic organ prolapse (POP) [1]. POP is defined as the descent of one or more of the pelvic organs from a normal position in the pelvis, resulting in a change of sensation, structure, or function [2]. A significant risk factor for PFD is childbirth [1]. However, many risk factors beyond childbirth influence the development of PFDs such as mode of delivery, parity, obesity, and age [3]. PFDs negatively impact women throughout the US and worldwide, including low-to-middle-income countries (LMICs).

With increased risk factors and the potential impact on physical, emotional, and sexual health, it is necessary to understand the prevalence of PFDs within low-income countries such as Uganda. Women may not seek care for PFDs owing to factors that may include embarrassment, misconception, lack of knowledge, social stigma, and decreased access to or the expense of specialized care. These factors can lead to underdiagnosis and undertreatment of PFDs.

Pelvic floor disorders can be determined by symptom assessment, administration of standardized questionnaires, and physical examination findings. Two valid and reliable instruments often used to assess the presence and impact of PFD on women are the Pelvic Floor Distress Inventory (PFDI) and the Pelvic Floor Impact Questionnaire (PFIQ). The short-form Pelvic Floor Distress Inventory (PFDI-20) has 20 questions across three categories: the Urinary Distress Inventory 6 (UDI-6), the Colorectal-Anal Distress Inventory 8 (CRADI-8), and the Pelvic Organ Prolapse Distress Inventory 6 (POPDI-6). The PFDI-20 is used to determine the presence of pelvic floor disorders and the degree to which symptoms bother the patient [4]. The short-form Pelvic Floor Impact Questionnaire (PFIQ-7) has seven questions to assess PFD [4]. This form is used to determine the presence of PFDs and their impact on the patient's life.

The PFDI-20 and PFIQ-7 have been validated in the English language [5]. Other studies have found the forms to be valid and reliable in African languages such as Tigrigna, Afrikaans, and Sesotho [6, 7]. To our knowledge, there are currently no valid instruments in Uganda to assess PFDs. The purpose of this study was to translate the English PFDI-20 and PFIQ-7 into Luganda, one of the major languages in Uganda, and to test the reliability and validity of these instruments in a cohort of parous Luganda-speaking women.

## Materials and Methods

Women who were enrolled in a pre-existing study were offered co-enrollment in this study. Written informed consent was obtained through bilingual study counselors. Data were collected from June 2022 to June 2023. Demographic data were obtained. The presence of PFD was measured by a standard symptom assessment and a standard physical examination. The Pelvic Organ Prolapse Quantification (POP-Q) examination was used to determine the presence of POP and a cough-stress test (CST) was used to measure the presence of stress urinary leakage. Translated versions of the PFDI-20 and PFIQ-7 were administered. Training was provided to all study personnel on both interview and examination techniques to ensure accuracy and sensitivity to participants’ cultures and values during a mandatory orientation.

Inclusion criteria were women at least 18 years old who were able and willing to provide informed consent, living within the study catchment area with no plans of relocation, able to return for two study visits, and willing and able to have a pelvic examination. Exclusion criteria included women who had recently undergone treatment for PFDs, women unwilling/unable to complete a pelvic examination, and women unwilling/unable to return for one follow-up visit.

The study was reviewed and approved by appropriate institutional review boards. The chronology of the study was translation, data collection visit 1, data collection visit 2, and validation.

### Translation

The PFDI-20 and PFIQ-7 surveys in English have cultural implications that may not apply to all cultures, particularly in the population of Ugandan women enrolled. The translation process was done in multiple stages to ensure that cultural and content implications were addressed. The process of translation focused on conceptual content rather than on word-by-word translation.

The first stage was a forward translation of the PFDI-20 and PFIQ-7 from English to Luganda by two bilingual English–Luganda translators in which Luganda is their primary language. These translated versions were compared and reconciled yielding Version 1 of the PFDI-20 and PFIQ-7. Version 1 of each questionnaire was administered to 10 Luganda-speaking individuals through a cognitive interview process with modifications made. Study staff combined these modifications to form Version 2 of each questionnaire. Version 2 was then back-translated into English. The English version was compared with the original and was reviewed by native English-speaking experts in the field of PFD to ensure accuracy. Once accuracy was ensured, the final versions of the PFDI-20 and PFIQ-7 were administered to the cohort of women.

The final versions had modifications that were made to ensure cultural accuracy and sensitivity (see Table 3 as an example).

### Validation

Evaluation of internal consistency (the degree of inter-relatedness among the items in a multi-item questionnaire measure) per the PFDI-20 and PFIQ-7 subscales was performed. Internal consistency was assessed using Cronbach’s α co-efficient. Test–retest reliability (the ability of the scores of an instrument to be reproducible if it is used on the same patient while the patient’s condition has not changed) was evaluated by repeat administration of the final version of the PFDI-20 and PFIQ-7 in patients at a 6-month interval. The questionnaires were administered at visit 1 at 0 months and again at visit 2 within 4–8 months of visit 1. Test–retest reliability of individual items and questionnaire subscale scores were calculated using weighted kappa (κ) correlations and intra-class correlation coefficients (ICCs).

Construct validity (the degree to which scores on the questionnaire measure relate to other measures: patient reports or clinical indicators) was evaluated by comparison of the PFDI-20 and PFIQ-7 results with patient symptom evaluation and physical examination as there are no other validated instruments in the population with which to compare. Spearman’s correlation coefficients and Wilcoxon rank sum tests were used to compare the severity of symptoms and POP according to PFDI-20 and PFIQ-7 responses and the POP-Q system respectively. Barber et al. and Kaplan et al. found the Spearman correlation coefficient between POP stage and survey responses to be 0.33 and 0.387 respectively [5, 8]. Assuming that the true correlation is 0.33, we have 99.0% power to detect a relationship between the two measurements, given our sample of 159 women [9].

Additionally, the known group validity of the questionnaires was assessed by comparing the PFDI-20 and PFIQ-7 responses of women with and without PFDs. This comparison was performed using a Wilcoxon rank sum test and a significant difference between the groups provides evidence that the questionnaires were adequately capturing the difference in symptoms between these two groups.

## Results

A total of 159 women were enrolled. The study was conducted from June 2022 to June 2023. The median age of participants was 35 (IQR 32–37). The median BMI, parity, and age at first delivery were 29.0 (IQR 24.3–33.3), 4 (IQR 3–5), and 19 (IQR 17–21) respectively. The majority of participants were married (47%) and worked as merchants (30%). Most participants had in-hospital (85%) vaginal (57%) deliveries. Most deliveries were reported as un-instrumented (96%) with a median weight of the largest infant being 3.80 kg (IQR 3.50–4.43). See Table 1 for demographic data.

**Table 1:** Demographics of parous Ugandan women

	*N* = 159
Age at first delivery (years)	19 (IQR 17, 21)
Occupation	
Farmer	16 (10%)
Homemaker	2 (1.3%)
Cook	17 (11%)
Manual laborer	1 (0.6%)
Merchant	48 (30%)
Unemployed	33 (21%)
Other	42 (26%)
Marital status	
Married	75 (47%)
No regular partner	2 (1.3%)
Regular partner	68 (43%)
Separated	10 (6.3%)
Divorced	1 (0.6%)
Widowed	3 (1.9%)
How many times has the patient been pregnant (gravity)?	4 (3, 6)
How many times has the patient given birth (parity)?	4 (3, 5)
Mode of delivery	
Only vaginal deliveries	91 (57%)
Only cesarean deliveries	21 (13%)
Both vaginal and cesarean deliveries	47 (30%)
Location of deliveries	
Only home deliveries	1 (0.6%)
Only hospital deliveries	135 (85%)
Both home and hospital deliveries	23 (14%)
Instrumented delivery	
Neither	153 (96%)
Vacuum	5 (3.1%)
Forceps	1 (0.6%)
Laceration at the time of delivery	
Unknown	21 (13%)
None	57 (36%)
1st degree	34 (21%)
2nd degree	45 (28%)
3rd degree	2 (1.3%)
4th degree	0 (0%)
Weight of largest infant (kg)	3.80 (IQR 3.50, 4.43)
BMI	29.0 (IQR 24.3, 33.3)

*IQR* interquartile range, *BMI* body mass index

As defined by symptom assessment, the prevalence of UI, AI, and POP at baseline were 28%, 0%, and 0% respectively. As defined by the POP-Q examination 93 participants (58.3%) had stage II POP or higher. When looking specifically at participants with stage II POP (91, 57%), 73 participants (80%) had POP above the hymen, 10 participants (11%) had POP that reached the hymen, and 8 participants (8.8%) had prolapse beyond the hymen. As defined by a positive CST, 72 participants (46%) had UI with an average bladder volume of 150 ml.

Administration of the translated forms of the PFDI-20 and PFIQ-7 demonstrated minimal bother or impact on activities of daily living. The median response to the PFDI-20 was 17 (IQR 0–31) and to the PFIQ-7 the median was 0 (IQR 0–0), revealing minimal bother or impact on quality of life by PFD respectively. The results of the PFDI-20 and PFIQ-7 questionnaires from the first administration are presented in Table 2.

**Table 2:** Prevalence of pelvic floor disorders (*PFDs*) in parous Ugandan women as determined by the Pelvic Floor Impact Questionnaire 7 (*PFIQ-7*) and Pelvic Floor Distress Inventory 20 (*PFDI-20*)

	*N* = 159
PFD	44 (28%)
Do you experience loss of urine beyond your control (urinary incontinence)?	44 (28%)
If yes: with what frequency does this occur?	
Less than once a month	16 (36%)
More than once a month	20 (45%)
More than once a week	7 (16%)
More than once a day	1 (2.3%)
Do you feel something bulging from the vaginal opening (prolapse)?	0 (0%)
Do you experience loss of stool (liquid or solid) beyond your control (fecal incontinence)?	0 (0%)
If yes: does this occur once per month?	
Yes	0 (NA%)
No	0 (NA%)
(Missing)	159
Scale score POPDI	0 (IQR 0, 8)
Scale score CRADI-8	0 (IQR 0, 13)
Scale score UDI-6	0 (IQR 0, 17)
PFDI-20 summary score	17 (IQR 0, 31)
UIQ-7	0.00 (IQR 0.00, 0.00)
CRAIQ-7	0.00 (IQR 0.00, 0.00)
POPIQ-7	0.00 (IQR 0.00, 0.00)
PFIQ-7	0.00 (IQR 0.00, 0.00)
POP-Q stage	
1	66 (42%)
2	91 (57%)
3	2 (1.3%)
Prolapse to or beyond hymen	20 (13%)
Cough Stress Test	
Positive	72 (46%)
Negative	86 (54%)
Volume of urine (ml)	150 (IQR 85, 258)

*POPDI* Pelvic Organ Prolapse Distress Inventory, *IQR* interquartile range, *CRADI-8* Colorectal-Anal Distress Inventory 8, *UDI-6* Urinary Distress Inventory 6, *NUIQ-7* Urinary Impact Questionnaire 7, *CRAIQ-7* Colorectal-Anal Impact Questionnaire, *POPIQ-7* Pelvic Organ Prolapse–Impact Questionnaire, *POP-Q* Pelvic Organ Prolapse Quantification

Internal consistency (the degree of inter-relatedness among the items in a multi-item questionnaire measure) assessed by Cronbach’s α co-efficient for the PFDI-20 and PFIQ-7 were α = 0.602 (95% CI 0.494–0.690) and α = 0.368 (95% CI −0.02 to 0.601) respectively. This suggests acceptable internal consistency for the Luganda version of the PFDI-20 and non-acceptable internal consistency for the PFIQ-7. Note that there were few positive responses on the PFIQ-7.

Test–retest reliability of individual items and questionnaire subscale scores was calculated using weighted kappa (κ) correlations and intra-class correlation coefficients (ICCs). The weighted κ for the PFDI-20 was 0.110 (95% CI 0.054–0.166) and the weighted κ for the PFIQ-7 was 0.039 (95% CI −0.056 to 0.133), suggesting slight agreement between participants’ initial and follow-up responses to the Luganda version of the questionnaires.

By comparing PFDI-20 subscales, construct validity was evaluated. UDI-6 scores were shown to have a strong positive association with the presence of urinary incontinence (Wilcoxon test *p* < 0.001), and the frequency of urinary incontinence (Spearman’s *r* = 0.386, *p* = 0.005). However, POPDI-6 scores were not shown to have a significant association with POP-Q stages being 2 or higher (Wilcoxon test *p* = 0.577). PFIQ-7 subscales were not analyzed in this manner, as there were few positive responses.

Spearman’s correlation coefficients were used to compare the severity of symptoms and POP stage according to PFDI-20 and PFIQ-7 responses and the POP-Q system respectively. The Spearman’s correlation coefficient between the Luganda prolapse assessment on the PFDI-20 and PFIQ-7 suggests a positive association (*r* = 0.252, *p* value < 0.001). However, we do not have evidence that suggests that the prolapse assessment on the PFDI-20 or the PFIQ-7 might be associated with stage of prolapse on POP-Q examination (*r* = 0.033, *p* value 0.683 and *r* = 0.006, *p* value 0.938 respectively).

When comparing the responses on the Luganda version of the PFDI-20 and PFIQ-7 in women with and without PFD using the Wilcoxon rank sum test we found the following. In the presence of PFD as defined by responses to symptom assessment, the Luganda PFDI-20 and PFIQ-7 seem to adequately capture the difference between groups (*p* value < 0.001). If the presence of PFD is defined based on stage II POP or higher on POP-Q examination, the PFDI-20 and POPDI do not seem to capture the difference between groups (*p* values 0.2 and 0.6 respectively). When the presence of PFD is defined as positive CST, again, the PFDI-20 and UDI-6 do not seem to capture the difference between groups (*p* value 0.6 and 0.6 respectively).

## Discussion

The PFDI-20 and PFIQ-7 are two valid and reliable instruments used to assess the presence of, degree of bother from, and impact of PFD on women. Women in low-income countries may be at an increased risk for PFD and it is necessary to provide reliable and valid surveys to assess these disorders. There is currently no known instrument to assess for the impact of PFD in Uganda, particularly in the Luganda language.

Many studies have assessed the reliability and validity of translating the English-language surveys into other languages, including Spanish, French, Chinese, and other African languages, such as Tigrigna, Afrikaans, and Sesotho [6, 7, 10]. Using methods like these studies with translation and back-translation, test–retest reliability, construct validity, and internal consistency, the PFIQ-7 and PFDI-20 were translated into the Luganda language and evaluated.

The weighted κ analysis shows that the PFDI-20 and PFIQ-7 are reliable based on the test–retest reliability, but only the PFDI-20 is reliable based on the internal consistency. This finding is due to minimal positive responses on the PFIQ-7; thus, the reliability could not be tested in this survey for internal consistency. Therefore, the PFDI-20 was found to be reliable in the Luganda language based on internal consistency. The PFIQ-7 could not be fully evaluated for reliability owing to the lack of positive responses.

When analyzing construct validity, the UDI-6 subscore of the PFDI-20 was found to have a strong association between the presence and frequency of UI. The POPDI-6 did not have a significant association between the presence of POP stage II or higher. The Wilcoxon analysis revealed that the way in which PFD is defined impacts the ability of the surveys to determine a difference between women with and without PFD. Both the PFDI-20 and PFIQ-7 show a difference between women with and without PFD if symptom assessment is how PFD is defined (*p* < 0.001). However, when defined as POP-Q stage of II or higher or as a positive CST, the PFDI-20 could not show a significant difference between the two groups. Therefore, the PFDI-20 and PFIQ-7 are valid measures when comparing symptom assessment between women with and those without PFD, but may not be valid based on how PFD is defined.

Overall, there is limited ability to determine the reliability and validity of the PFDI-20 and PFIQ-7 owing to a low positive response rate in our cohort. The UDI-6 portion of the PFDI-20 in the Luganda language is considered a reliable and valid test, and the PFIQ-7 is considered valid when comparing symptoms between women with and without PFD. This limitation may be a result of the low prevalence of PFD in the population rather than a limitation of the Luganda versions of the PFDI-20 and PFIQ-7.

In the study evaluating reliability and validity in the US by Barber et al., PFDI-20 and PFIQ-7 were considered reliable and valid [5]. Henn et al. showed the translation of PFDI-20 and PFIQ-7 into the African languages of Afrikaans and Sesotho to be reliable and valid [7]. The validity and reliability differences between this study and previous translation studies is likely due to prevalence differences, which may be the result of age differences. With a younger cohort at a median age of 35, fewer women had symptomatic POP than in other studies with older cohorts. This difference impacts the study, as the surveys screen for impact of PFD on life and if most women do not have symptomatic PFD, they will screen negative as seen in this study, particularly using the PFIQ-7. A large portion of women with prolapse determined by POP-Q examination had prolapse that is above the hymen and most women with prolapse more than 0.5 cm above the hymen rarely report symptoms [11]. This may be a reason why women with prolapse were asymptomatic.

Additionally, the cohort of women surveyed may have limited knowledge of PFDs or perhaps consider their presence to be normal. This study found that women with a positive CST did not have a statistically significant degree of bother according to the surveys. This could suggest that participants might consider stress incontinence to be a normal part of life for women or not viewed as bothersome. However, without further analysis, this interpretation cannot be fully determined.

Another difference between the US surveys and the Luganda surveys is that the Luganda surveys were administered by study staff whereas the US surveys are generally self-administered. This approach was necessary, as not every participant was able to read in Luganda and having study staff administer prevented inaccurate results due to illiteracy. This technique is likely going to be a necessity in many LMICs with surveys. Responding to study staff rather than self-administered participation may impact how forthcoming participants are regarding PFD.

Another difference compared with other translations of the PFIQ-7 was that the Luganda version had semantic changes, as shown in Table 3. The semantic changes in the Luganda version were necessary to be culturally competent, as some of the activities in the English-language form could not be applied to women in Uganda. The conceptual translation did not change the validity of the PFIQ-7, as it still assessed for symptoms comparing people with and without PFD. This is notable, as allowing for conceptual translations to be used in other languages and countries will bring further access to the PFIQ-7.

**Table 3:** Changes in the Luganda version of the Pelvic Floor Impact Questionnaire 7 (*PFIQ-7*) as compared with the English-language validated surveys

Question number	English PFIQ-7	English translation of the Luganda PFIQ-7
2	“Ability to do physical activities such as walking, swimming or other exercise?”	“Ability to do physical activities such as walking or carrying heavy loads?”
3	“Entertainment activities such as going to a movie or concert?”	“Entertainment activities such as attending a function, dancing, or going to church?”
6	Emotional health (nervousness, depression, etc.)?	“Emotional health (nervousness, lack of concentration, sleep disturbance, loss of appetite, fear, anxiety, etc.)?”

A strength of this study was that 159 participants responded to both the original and the retested survey (no loss to follow-up). Every study participant was assessed twice over 4–8 months. The data were prospectively collected. Both symptom assessment and standardized physical examinations were completed. Another strength was that each survey was administered by native Luganda-speaking study staff for both visits, allowing for all individuals to participate, regardless of literacy.

A limitation of this study is the age range of participants as the median age was 35. As women age, pelvic floor disorders become more common. Despite being parous, the younger cohort of women likely led to a lower prevalence of symptomatic PFDs. Another limitation is the generalizability of our findings to the population of Uganda. This study was set in a pre-existing cohort of women (parous) in a specific region of Uganda. Last, the survey was administered by a staff member, which could impact the responses of women and could also be a limitation of the results presented. However, with reliable and valid surveys in the Luganda language, these surveys could be used to assess for PFDs in Luganda-speaking women throughout the country and world.

In the future, this study should be expanded to a larger population of Luganda-speaking women that have a higher prevalence of and severity of PFD, likely an older cohort of women. It is known that age increases the risk of PFDs, so expanding to a cohort with a higher median age would help to better estimate prevalence and help to further determine the reliability and validity of these surveys. Additionally, the Luganda version of the PFDI-20 and PFIQ-7 could be further refined through an iterative process with women who have known PFD.

## Conclusion

The UDI-6 portion of the PFDI-20 was found to be valid in the Luganda language. The inability to determine if the PFIQ-7 and entirety of the PFDI-20 were reliable and valid is likely due to the relatively low prevalence of PFD in our younger population. These surveys should be further tested in a larger population with a higher prevalence of advanced PFD. Most participants had POP that did not cross the hymen, which has been found to cause minimal bother to the women studied. This study showed that the translation process to more languages in Uganda and other African countries is possible and necessary to begin to provide access to PFD care.

## Data Availability

The data supporting these findings is available on request to the corresponding author.
